# Multivariate NTCP Model of Hypothyroidism After Intensity-Modulated Radiotherapy for Nasopharyngeal Carcinoma

**DOI:** 10.3389/fonc.2021.714536

**Published:** 2021-08-23

**Authors:** Guanzhu Shen, Yinglin Peng, Jian Li, Haijun Wu, Guangshun Zhang, Chong Zhao, Xiaowu Deng

**Affiliations:** ^1^Department of Radiation Oncology, State Key Laboratory of Oncology in South China, Collaborative Innovation Center for Cancer Medicine, Sun Yat-sen University Cancer Center, Guangzhou, China; ^2^Department of Radiation Oncology, The Third Affiliated Hospital of Sun Yat-sen University, Guangzhou, China; ^3^School of Biomedical Engineering, Sun Yat-sen University, Guangzhou, China; ^4^Department of Radiation Oncology, Central Hospital of Guangdong Nongken, Zhanjiang, China; ^5^Department of Radiation Oncology, Cancer Center, First People’s Hospital of Foshan, Affiliated Foshan Hospital of Sun Yat-sen University, Foshan, China

**Keywords:** nasopharyngeal carcinoma, intensity-modulated radiotherapy, hypothyroidism, EQD2, NTCP mode

## Abstract

**Objective:**

To evaluate the incidence of hypothyroidism in patients with nasopharyngeal carcinoma after intensity-modulated radiotherapy (IMRT), analyze its correlation with multiple influencing factors such as thyroid exposure dose, thyroid volume, and gender, and construct a multivariate-based normal tissue complication probability (NTCP) model for the occurrence of hypothyroidism after IMRT.

**Materials and Methods:**

The thyroid hormone levels of patients at different points in time before and after radiotherapy were tested, and statistics on the incidence of hypothyroidism after treatment were obtained. The dose-volume data of patients’ thyroids were converted into EQD2 equivalent dose values. The correlation between hypothyroidism after radiotherapy and thyroid exposure dose, thyroid volume, gender, and other factors was analyzed, and an NTCP model was constructed.

**Results:**

A total of 69 patients with nasopharyngeal carcinoma were enrolled in this study. Twelve months after radiotherapy, a total of 24 patients (34.8%) developed hypothyroidism. Univariate analysis and multivariate analysis revealed that the average thyroid dose and thyroid volume are the most important factors affecting hypothyroidism after radiotherapy. The NTCP model constructed based on the average dose and thyroid volume has a good degree of fit.

**Conclusion:**

The volume and average dose of the thyroid gland are the key factors affecting the occurrence of hypothyroidism in patients with nasopharyngeal carcinoma after radiotherapy. The NTCP model constructed based on multivariate construction suggests that reducing the average dose of the thyroid to the greatest extent is an effective way to protect thyroid functions.

## Introduction

Radiotherapy-induced hypothyroidism (RHT) is one of the common late-stage toxic reactions in patients who have received cervical radiotherapy, and its incidence is as high as 20%–40% (1–3), which is higher than the incidence in the normal population. The occurrence of hypothyroidism after radiotherapy in most studies was concentrated within 5 years after radiotherapy, and it generally reached a peak approximately 1 to 3 years after radiotherapy (4–6).

Intensity-modulated radiation therapy (IMRT) is the main treatment for nasopharyngeal carcinoma. Notably, 70% to 80% of patients with nasopharyngeal carcinoma had cervical lymph node metastasis at the first diagnosis (7, 8), and prophylactic irradiation of the neck lymph node drainage area is inevitable. The thyroid is located in the prophylactic irradiation area of the neck and is exposed to higher doses of radiation. However, there are currently few reports on the incidence of hypothyroidism in nasopharyngeal cancer patients after IMRT. Huang et al. retrospectively analyzed the data of 98 nasopharyngeal cancer patients who received IMRT. The lower neck prophylactic irradiation area was given a prescribed dose of 54 Gy. The median follow-up period was 17 months, and the results showed that the average thyroid dose was 49.72 Gy, and the incidence of hypothyroidism was 33.7% (9). Hypothyroidism due to radiotherapy generally has an insidious onset and lacks typical symptoms and signs; therefore, it is often ignored by clinicians and patients.

Gender (10–12), age (5, 13, 14), and thyroid volume (10, 15) are factors that affect hypothyroidism after radiotherapy, but thyroid exposure dose is the most important factor (11, 16). The incidence of hypothyroidism increases with the exposure dose, and its specific threshold is still inconclusive. Kim et al. reported the follow-up results of 114 patients with head and neck tumors after radical radiotherapy. V45 = 50% is the threshold for hypothyroidism (17), and subsequent meta-analysis results additionally support this conclusion (18). However, some studies believe that V30 or V50 is the dose threshold for hypothyroidism (19–21).

The equivalent dose in 2 Gy fractions (EQD2) is the dose required to achieve the same biological effect as conventional fractionated radiotherapy with fractions of 2 Gy. IMRT can deliver different fractionated doses of radiation to target areas and organs at risk in the irradiation field. In the treatment of nasopharyngeal carcinoma using the simultaneous measurement technique, the fractionated dose of the primary tumor target area often exceeds the conventional fractions of 2 Gy/time, and the fractionated dose of organs at risk is often lower than that of conventional fractionation. However, the dose tolerance value of each organ at risk is calculated based on the biological effects of fractionated irradiation with 2 Gy fractions of conventional radiotherapy. It is impossible to accurately assess the biological effects and probability of possible damage depending on the physical dose for organs-at-risk obtained using an IMRT treatment planning system (TPS) alone. The thyroid is a late-reacting tissue and is greatly affected by fractionated doses. Therefore, in the case of IMRT, the threshold value of the dose-volume factor based on the physical dose cannot be a reference for the limited conditions of the plan design. Additionally, it is impossible to select a reasonable IMRT dose limit level directly based on the relationship between the thyroid injury probability and the dose-volume under conventional fractionated irradiation.

This study aims to assess the incidence of hypothyroidism after radiotherapy in patients with nasopharyngeal carcinoma, analyze the correlation between hypothyroidism after radiotherapy and possible influencing factors, and construct a normal tissue complication probability (NTCP) model for the occurrence of hypothyroidism after IMRT, based on the equivalent dose in 2 Gy fractions (EQD2). In addition, this model is used to guide the search for more reasonable conditions for thyroid dose optimization in IMRT.

## Materials and Methods

### Case Screening

All patients were pathologically diagnosed with nasopharyngeal carcinoma and received nasopharyngeal + cervical IMRT at Sun Yat-sen University Cancer Center. No distant metastasis was observed in the first diagnosis. Further, there was no history of hyperthyroidism, thyroiditis, and other thyroid-related diseases before treatment and no history of thyroid surgery, as well as no medical or surgical history corresponding to pituitary-related diseases. Moreover, the thyroid functions [including thyroid stimulating hormone (TSH)] were normal before treatment, and regular follow-up and thyroid function tests were performed as required after the treatment.

Before treatment, and three months and twelve months after treatment, venous blood was drawn from the patients *via* the electrochemiluminescence method for thyroid function tests. The test items include free triiodothyronine 3 (fT3), free triiodothyronine 4 (fT4), TSH, and thyroid peroxidase antibody. The clinical diagnosis of hypothyroidism depends on the related symptoms and signs of hypothyroidism, the extent to which the serum TSH is higher than the normal upper limit, and the extent to which fT3 and fT4 are lower than the normal lower limit. If the patient has no clinical manifestations, but the TSH in the blood circulation is higher than the normal upper limit with or without fT3 and fT4 level abnormalities, the patient can be diagnosed with subclinical hypothyroidism.

### Intensity-Modulated Radiotherapy and Dose Conversion

The target area delineation method and prescription dose administration of IMRT are described in our previously published literature (22). The treatment planning system (TPS) used Eclipse 11.0. The thyroid delineation entailed a layer-by-layer delineation of the thyroid structure based on enhanced CT scan images. The delineation range included the entire left and right lobes and isthmus of the thyroid, but no restriction was set for the thyroid dose.

The patients’ IMRT regimen was exported from the TPS to an EQD2 calculation and evaluation software in DICOM RT format. The calculation software automatically calculated the pixel-by-pixel conversion of EQD2 according to the set thyroid α/β value and the mature linear quadratic model calculation formula of radiobiology, and reconstructed the relevant EQD2 dose-volume parameters.

The EQD2 calculation formula is as follows: $EQD2=D\frac{d+\alpha /\beta }{2+\alpha /\beta }$

where D is the total dose and d is the fractionated dose. We consulted reports in authoritative literature (23) and set the α/β value of the thyroid gland to be 3 Gy. The overlap between the thyroid and PTV2 was calculated according to the thyroid α/β value.

Data collection included the maximum thyroid dose Dmax, minimum dose Dmin, average dose Dmean, fractionated dose, V10 (percentage of thyroid volume where the exposure dose exceeded 10 Gy), V20, V30, V40, V45, V50, V60, V70, and thyroid volume after conversion to EQD2.

### Data Analysis

SPSS 16.0 statistical analysis software was used for the analysis. Patients were divided into a hypothyroidism group and normal thyroid function group according to whether hypothyroidism occurred 12 months after treatment. If Dmean, age, and thyroid volume between the two groups conformed to the condition of normal homogeneity of variance, an independent sample T test was performed; otherwise, the rank sum test of independent samples was used, and the chi-square test was used for the comparison of categorical data such as gender, staging, and other factors. The thyroid hormone levels at different points in time (if they conformed to the normal homogeneity of variance) were analyzed by repeated measures analysis of variance and pairwise tests, and p < 0.05 was considered to indicate statistical significance.

According to whether hypothyroidism occurred 12 months after treatment, factors that may affect thyroid function, including gender, age, T stage, N stage, clinical stage, thyroid volume, Dmax, Dmin, Dmean, fractionated dose and V10 to V70, were analyzed by logistic regression analysis, one by one, to select independent influencing factors with statistical significance. Spearman correlation analysis was used to analyze the correlation between various factors, and representative factors were selected to perform logistic regression multi-factor analysis, by the forward method, to select the factors affecting the occurrence of thyroid function after radiotherapy and derive the logistic regression equation through fitting.

### NTCP Model Construction

The NTCP model equation determined the mixture model for calculation (24, 25); the formula is as follows.

$$\text{NTCP}={\left(1+e^{-s}\right)}^{-1}$$

where *S* is the logistic regression equation derived through the fitting with factors selected by multi-factor analysis.

## Results

### General Data of Patients

From August 2012 to January 2014, a total of 69 patients met the enrollment requirements and entered the study. Among them, 53 were males and 16 were females, with a median age of 43 years (11–64 years). The general data of all patients are shown in **(** **Table 1** **)**.

**Table 1:** Patient and treatment characteristics.

	No.	Percentage
Gender		
Male	53	76.8
Female	16	23.2
Age	43 (11–64) years old	
T stage*		
T1	5	7.3
T2	17	24.6
T3	35	50.7
T4	12	17.4
N stage*		
N0	11	15.9
N1	29	42.0
N2	27	39.1
N3	2	2.9
Clinical stage*		
I	2	2.9
II	17	24.6
III	38	55.1
IV	12	17.4
Treatment method		
Radiotherapy alone	11	15.9
Radiotherapy+chemotherapy	58	84.1

*According to the AJCC 7^th^ Edition staging system.

### Changes in Thyroid Hormone Levels After Radiotherapy Compared to Those Before Radiotherapy

**Figure 1:**
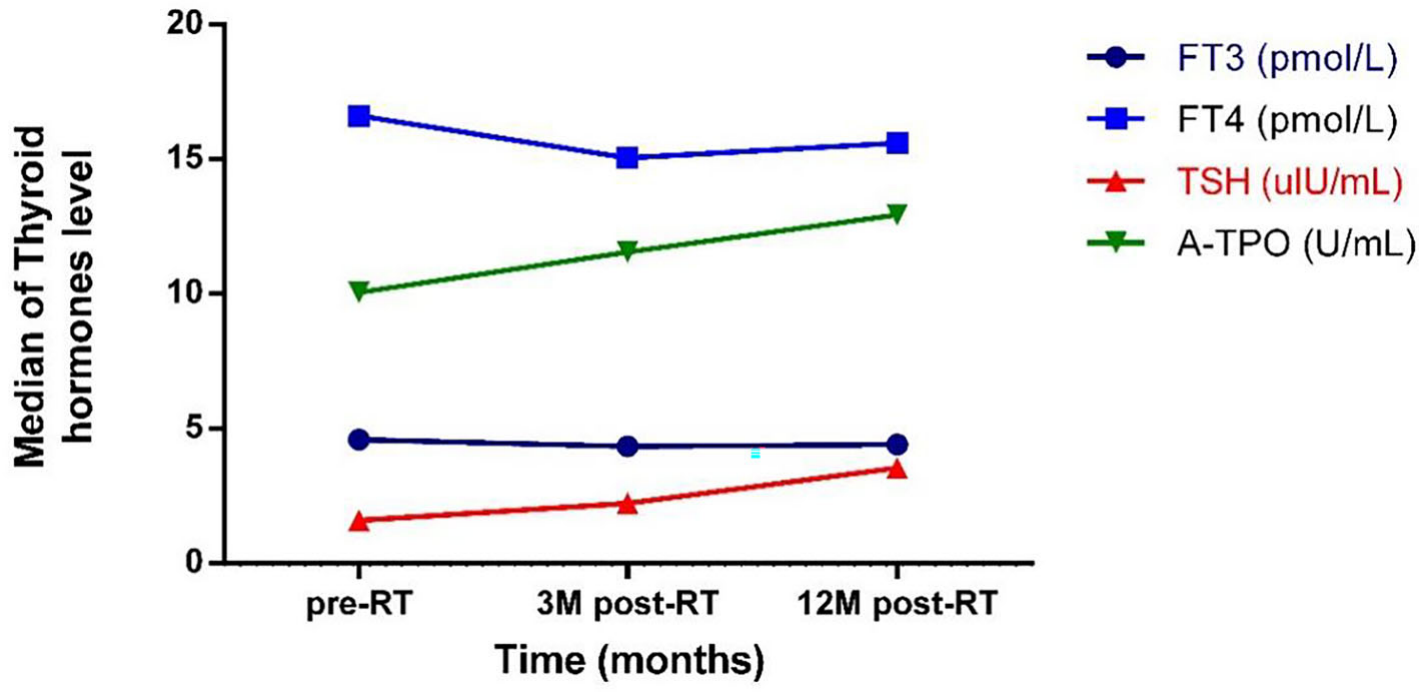
The trend of the change of thyroid hormones level with time. pre-RT, previous radiotherapy; 3M post-RT, 3 months post radiotherapy; 12M post-RT, 12 months post radiotherapy; FT3, Free triiodothyronine 3; FT4, Free triiodothyronine 4; TSH, Thyroid stimulating hormone; A-TPO, A-Thyroid peroxidase.

There was no significant difference in serum fT3 levels between different points in time. Twelve months after radiotherapy, the level of fT4 was significantly lower than that before radiotherapy (p < 0.001). The TSH level showed a continuous upward trend from before to after radiotherapy (pairwise comparison showed each p < 0.001). Twelve months after radiotherapy, the level of A-Thyroid peroxidase (A-TPO) was significantly higher than that before radiotherapy (p = 0.011) **(** **Figure 1** **)**.

### Thyroid Dose and Volume

After converting the thyroid physical dose into EQD2, the median value of Dmin of the whole group was 21.34 ± 10.71 Gy (0.80 to 35.69 Gy); the median value of Dmax was 63.89 ± 4.39 Gy (51.42 to 74.89 Gy); the median value of Dmean was 41.79 ± 11.02 Gy (10.31 to 51.46 Gy); and the median value of fractionated dose was 1.37 ± 0.35 Gy (0.34 to 1.83 Gy).

**Figure 2:**
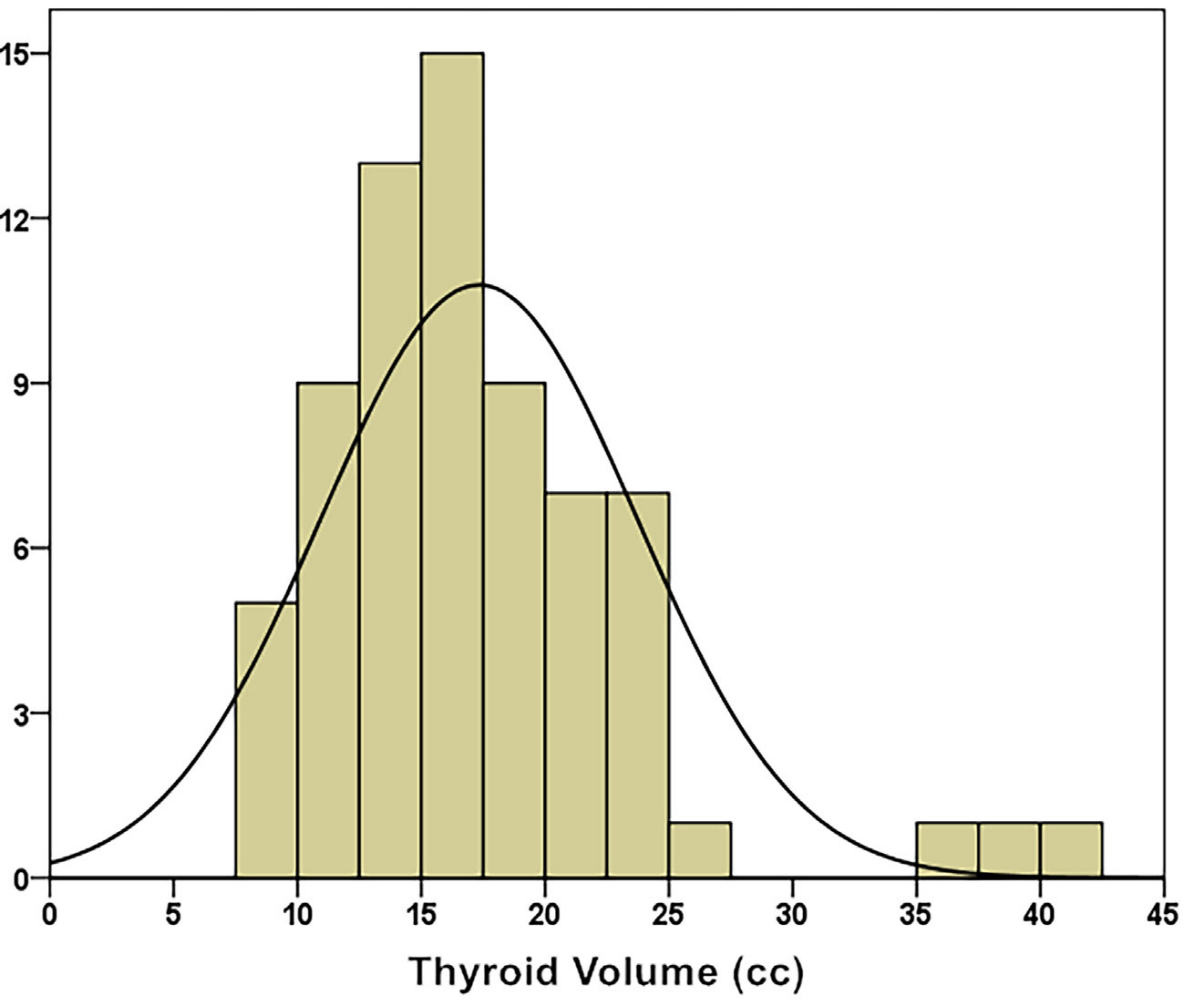
Thyroid volume distribution of 69 patients.

The median thyroid volume was 16.60 ± 6.38 cc (8.19 – 42.00 cc). Through the K–S test (Kolmogorov–Smirnov test), it was known that the thyroid volume distribution approximately conformed to a normal distribution (Kolmogorov–Smirnov Z = 0.968, p = 0.306), as shown in **(** **Figure 2** **)**.

### Hypothyroidism After Radiotherapy

The thyroid hormone levels of all patients before radiotherapy were within the normal range. Three months after radiotherapy, 6 patients developed hypothyroidism, the prevalence rate was 8.7%, of which 5 cases had subclinical hypothyroidism, including 3 males and 2 females. Additionally, there was a case of clinical hypothyroidism in a 45-year-old male patient. Twelve months after radiotherapy, there were 24 cases of abnormal thyroid function, including 2 cases of clinical hypothyroidism and 22 cases of subclinical hypothyroidism. The total incidence of hypothyroidism was 34.8%, of which 16 patients were male and 8 patients were female and both patients with clinical hypothyroidism were male. The patients were categorized into the hypothyroidism group (24 cases) and normal thyroid function group (45 cases) according to whether hypothyroidism occurred twelve months after radiotherapy, and the differences between the two groups were statistically analyzed. There was a significant difference in age between the two groups (median age: 39 years in the hypothyroid group *vs.* 45 years in the normal group, p = 0.005). There was no statistical difference in gender, T, N staging, and clinical staging.

The median value of Dmin in the two groups was 24.86 ± 7.92 Gy in the hypothyroidism group *vs.* 16.00 ± 10.65 Gy in the normal group (p = 0.001). The median values of Dmax were: 64.29 ± 4.61 Gy in the hypothyroidism group *vs.* 63.62 ± 4.31 Gy in the normal group (p = 0.681). The median value of Dmean was 46.54 ± 7.37 Gy in the hypothyroidism group *vs.* 38.44 ± 11.45 Gy in the normal group, (p = 0.001). The median thyroid volumes of the two groups were: 13.46 ± 3.86 cc (8.19 to 20.50 cc) in the hypothyroidism group *vs.* 17.10 ± 6.80 cc (8.80 to 42.00 cc) in the normal group (p = 0.001); the thyroid volume in the hypothyroidism group was significantly smaller than that of the normal thyroid function group.

### Logistic Univariate Analysis and Multivariate Analysis

Logistic univariate regression analysis revealed that age, Dmin, Dmean, V20 to V50, and thyroid volume were all related factors that affected the occurrence of hypothyroidism. Because there might be certain correlations between the above-mentioned related factors, Spearman correlation analysis was used to analyze the related factors that affected the occurrence of hypothyroidism, and Spearman coefficient > 0.8 was used as the criterion to select representative factors. The results showed that Dmean had a strong correlation with Dmin, V20 to V50 (Spearman correlation coefficients were all > 0.8), while age and thyroid volume had a relatively weak correlation with other factors.

The three independent factors of age, Dmean, and thyroid volume were analyzed by logistic multivariate regression analysis. The results showed that Dmean (p = 0.016) and thyroid volume (p = 0.011) were independent prognostic factors **(** **Table 2** **)**.

**Table 2:** Univariate analysis and multivariate analysis of radiation induced hypothyroidism.

Parameters	Univariate		Multivariate	
	Hazard ratio (95% CI)	P-value	Hazard ratio (95% CI)	P-value
Gender	2.312 (0.738-7.245)	0.15		
Age	0.934 (0.887-0.984)	0.01	0.957 (0.906-1.011)	0.12
T-classification	1.920 (0.973-3.789)	0.06		
N-classification	1.579 (0.830-3.107)	0.186		
Clinical stage	1.702 (0.820-3.531)	0.153		
Chemotherapy	6.571 (0.787-54.851)	0.082		
Fractionated dose	1.358 (0.869-2.123)	0.179		
Dmin	1.001 (1.000-1.002)	0.002		
Dmax	1.000 (0.999-1.001)	0.676		
Dmean	1.001 (1.000-1.002)	0.004	1.001 (1.000-1.002)	0.016
V10	1.046 (0.999-1.095)	0.053		
V20	1.046 (1.006-1.088)	0.023		
V30	1.038 (1.012-1.066)	0.004		
V40	1.046 (1.020-1.073)	0.001		
V45	1.046 (1.018-1.075)	0.001		
V50	1.052 (1.017-1.089)	0.004		
V60	1.056 (0.979-1.139)	0.156		
V70	0.580 (0.026-12.933)	0.731		
Volume	0.808 (0.703-0.928)	0.003	0.838 (0.721-0.975)	0.011

The logistic equation obtained after fitting is

S=−1.385+(0.093×Dmean)+(−0.188 × thyroid volume)

### Normal Tissue Complication Probability Model

According to the calculation method of the mixture model, the following NTCP calculation formula is obtained:

NTCP = (1 + *e^S^*)^–1^, where *S* = -1.385 + (0.093 × Dmean) + (-0.188 × thyroid volume), the unit of Dmean is Gy, and the unit of thyroid volume is cc. This equation has passed the goodness of fit test (Hosmer–Lemeshow: p = 0.698) and has a good degree of fit, indicating that the probability of hypothyroidism is positively correlated with the average thyroid dose, and it increases with an increase in the average thyroid dose (OR value = 1.098/Gy, 95% confidence interval of the OR value: 1.018 to 1.184). Contrastingly, the probability of hypothyroidism is negatively correlated with thyroid volume: as the thyroid volume decreases, the probability of hypothyroidism increases (OR value = 0.829/cc, 95% confidence interval of OR value: 0.717 to 0.958).

**Figure 3:**
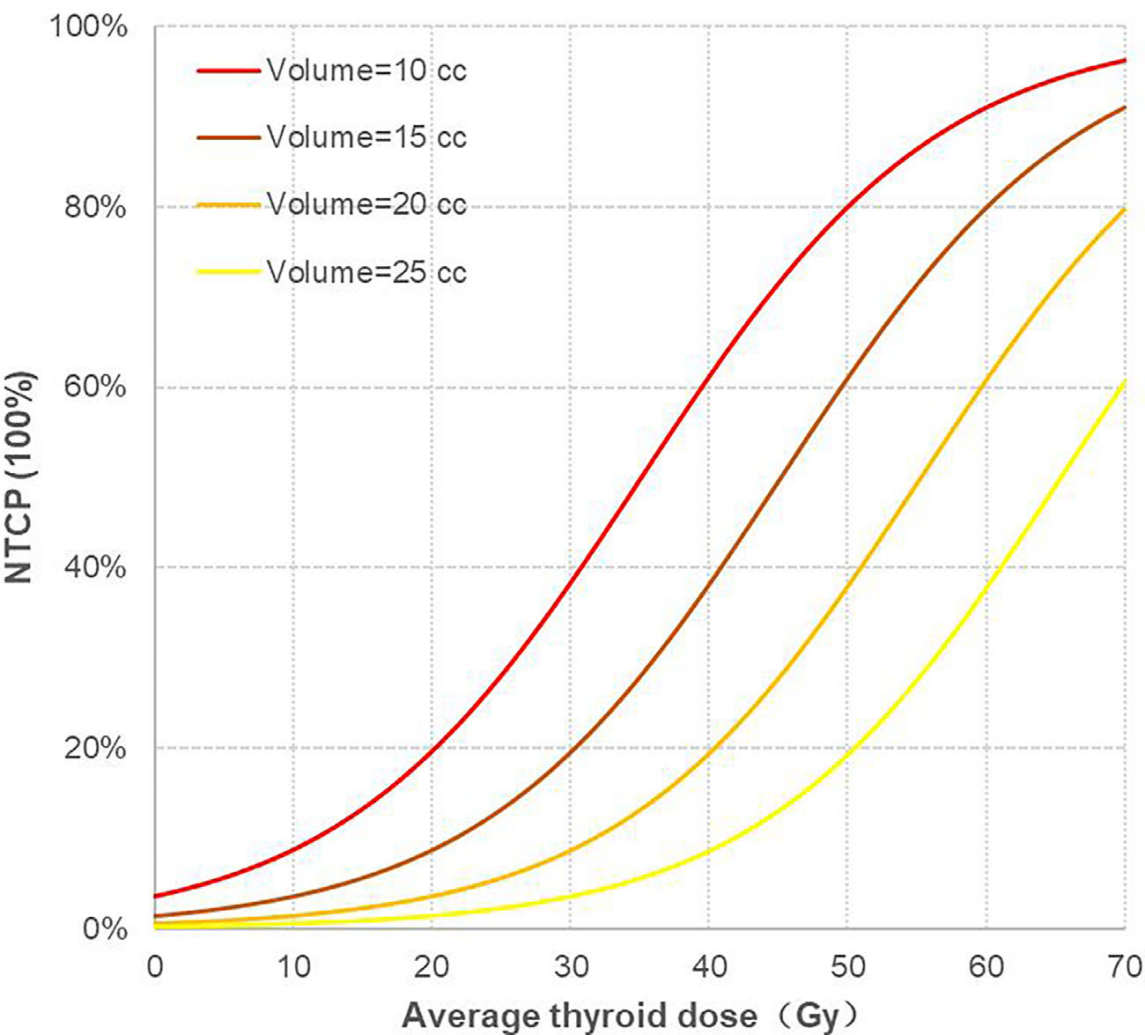
Corresponding NTCP curves of four different thyroid volume levels. NTCP, normal tissue complication probability.

In this study, the thyroid volume of 69 patients was mostly concentrated from 10 cc to 25 cc; thus, the thyroid volume was divided into four levels: 10 cc, 15 cc, 20 cc, and 25 cc, and the corresponding NTCP curves were drawn, as shown in **(** **Figure 3** **)**. There were two variables in the NTCP model, and the distribution of thyroid volume conformed to the normal distribution. Taking the median thyroid volume of 16.60 cc, the TD5/1 and TD10/1 of RHT were calculated as 16.67Gy and 24.77Gy, respectively.

## Discussion

Hypothyroidism is one of the common complications of head and neck tumors and nasopharyngeal cancer among patients after receiving cervical radiotherapy. Previous studies mostly involved patients who received radiotherapy for head and neck tumors. However, patients with head and neck tumors often underwent surgery before radiotherapy. The results of a meta-analysis show that patients who underwent hemithyroidectomy or surgery in the neck that did not involve the thyroid gland had a higher incidence of hypothyroidism (18). Radiotherapy is the main treatment for nasopharyngeal cancer, in contrast with the case of head and neck tumors. Previously, there were few reports of hypothyroidism in patients with nasopharyngeal carcinoma after radiotherapy. Wu et al. reported the occurrence of hypothyroidism in 408 patients with nasopharyngeal carcinoma who received conventional radiotherapy or three-dimensional radiotherapy; the incidence of hypothyroidism after 5 years of treatment was 24.7% (2). After the treatment of nasopharyngeal carcinoma entered the era of IMRT, the incidence of hypothyroidism has increased. Studies have shown that among nasopharyngeal carcinoma patients who received IMRT, 33.7% of patients had hypothyroidism at a median period of 17 months after radiotherapy (9); Zhai et al. reported that after patients with nasopharyngeal carcinoma received IMRT, the incidence of hypothyroidism after 2 and 3 years was 29.6% and 43.9%, respectively (26). In this study, 34.8% of patients who used IMRT had hypothyroidism 12 months after radiotherapy, which was close to the results of previous studies. The main reason for the significant increase in the incidence of hypothyroidism may be as follows: In IMRT, the radiation dose in the prophylactic cervical irradiation area, especially the metastatic cervical lymph nodes, is significantly higher than that of conventional radiotherapy to obtain a better area control rate, and the exposure dose of the thyroid, which is close to the prophylactic cervical irradiation area, is correspondingly increased; secondly, when conventional radiotherapy is used for prophylactic irradiation of the neck, a 3 cm wide lead shield is usually set in the front tangent field of the neck to protect the spinal cord. Therefore, the thyroid can be partially blocked by the lead shield, thereby avoiding exposure to higher doses of radiation.

In this study, it can be observed from the trend of hormone levels that the thyroid fT3 level decreased slightly after radiotherapy; the serum fT4 level decreased significantly, while the serum TSH level and A-TPO level continued to increase after treatment. This trend is consistent with the previous research results of Lin et al. (27). Studies have confirmed that thyroid autoantibodies are one of the important factors in the occurrence of hypothyroidism after radiotherapy (14, 28, 29). The study by Lin et al. found that the serum A-TPO level of nasopharyngeal carcinoma patients who had hypothyroidism after radiotherapy was significantly higher than that of patients with normal thyroid function, and the serum A-TPO level of patients was negatively correlated with fT4 levels (27). A study observed significant increases in thyroid inflammation-related indicators such as thyroid vascular pulsatility index and resistance index after radiotherapy under Doppler ultrasound, and it is believed that thyroid inflammation due to radiation damage is an important link in the occurrence of hypothyroidism after radiotherapy (30). Moreover, inflammation of the thyroid can stimulate the production of A-TPO, which may further aggravate the damage to the thyroid.

Since the nasopharynx is adjacent to the sphenoid bone and the pituitary fossa, the pituitary is often exposed to higher doses due to its proximity to the target area. Central hypothyroidism due to insufficient secretion of the trophic hormone by the pituitary gland is characterized by serum TSH levels that are lower than normal, with or without abnormal serum fT3 and fT4 levels. A recent report retrospectively analyzed 135 cases of hypothyroidism in nasopharyngeal carcinoma patients after IMRT treatment. The median follow-up period was 34.1 months. Notably, 28.9% (39/135) of the patients had primary hypothyroidism and no central hypothyroidism occurred (26), which is similar to the results reported by McDowell et al. (31). Additionally, there are many studies showing that the pituitary dose does not affect the occurrence of central hypothyroidism after radiotherapy, and the thyroid radiation dose is an influencing factor for the occurrence of primary hypothyroidism after radiotherapy (32, 33). In this study, only 2 patients in the entire group showed a continuous small drop in serum TSH levels three months and twelve months after radiotherapy compared with the levels before radiotherapy, but no patients had serum TSH levels lower than normal after radiotherapy, proving that no patients experienced the decompensation of pituitary functions.

In this study, the NTCP model of hypothyroidism constructed based on EQD2 showed that thyroid volume and average dose are two independent factors that affect the occurrence of hypothyroidism after radiotherapy, which is consistent with the results of multiple previous studies (15, 20, 24). However, some researchers tend to believe that a specific dose-volume value, such as VS60, V40, or V50, is a risk factor for hypothyroidism (5, 19, 34). The results of this study show that Dmean is one of the most important factors influencing the occurrence of hypothyroidism after radiotherapy, and its impact is greater than that of any of the indicators among V20 to V50, which additionally means that the occurrence of hypothyroidism is not affected only by the percentage of the volume of the thyroid that receives high doses of radiation. Because even if the percentage of thyroid volume exposed to high doses is very small, the overall thyroid exposure dose Dmean can still be high and increase the risk of hypothyroidism. This is in line with the relevant previous hypothesis about the mechanism of hypothyroidism after radiotherapy: that is, in addition to the damage to thyroid acinar cells, radiation can cause damage to the endothelial cells of thyroid nutrient vessels, leading to atherosclerosis, narrowing of the lumen, and insufficient blood supply to the thyroid gland (29, 30, 35, 36). In addition, thyroid inflammation due to radiotherapy leads to increased levels of thyroid autoantibodies, and autoimmune reactions between thyroid autoantibodies such as A-TPO and the thyroid may additionally play a damaging role in the occurrence of hypothyroidism (27, 37). Although the percentage of thyroid volume exposed to high doses is very small, local ischemia and the autoantibody immune response of thyroid can still affect the secretory function of the entire thyroid. Therefore, when evaluating IMRT plans for patients with nasopharyngeal carcinoma, simply using a certain dose-volume threshold such as V20 to V50 for the thyroid dose assessment may underestimate the risk of hypothyroidism, and it may be more reasonable to use Dmean for the assessment.

Thyroid volume is another key factor that affects the occurrence of hypothyroidism after radiotherapy. Studies have shown that for every 1 cc increase in the thyroid volume before treatment, the incidence of hypothyroidism can be reduced by 7% (6). Additionally, our study observed that there is a negative correlation between the thyroid volume and the occurrence of hypothyroidism, that is, the larger the thyroid volume before treatment, the lower the risk of hypothyroidism after radiotherapy. Thyroid volume is a protective factor for hypothyroidism after radiotherapy. which is consistent with the results of multiple previous studies (1, 6, 24).

The cases number met the enrollment criteria and entered this study were relatively small at the time, it was not able to create an adequate validation data set for external verification of the NTCP model. In addition, the time of follow-up was not long enough to assess the incidence of hypothyroidism at 3 or 5 years after radiotherapy. Muthy et al. (38) reported the hypothyroidism after radiotherapy in 122 patients with advanced head and neck tumors in a median follow-up time of 41 months, the peak of hypothyroidism occurred about 1 year after receiving radiotherapy (median time of subclinical hypothyroidism was 11.5 months, and median time of clinical hypothyroidism was 14.5 months). For the median thyroid volume of 16.60 cc, our study shown that the TD5/1 and TD10/1 of radiation induced hypothyroidism were 16.67Gy and 24.77Gy, respectively. Further study to expand and verify the model is undergoing and will be reported when enough cases are collected.

Overall, this study constructed an EQD2-based NTCP model of hypothyroidism after IMRT for nasopharyngeal carcinoma, which can provide an accurate basis for a more optimized thyroid dose restriction strategy. Accurately delineating the scope of the thyroid gland and regarding it as an organ at risk for dose restriction according to the NTCP model is the basis for reducing the occurrence of hypothyroidism. For a certain thyroid volume, the average thyroid dose should be reduced to the greatest extent. When selecting IMRT techniques, a multi-leave-collimator with a higher resolution should be used to maximize the dose conformity of the target area and increase the dose gradient between the radiotherapy target zone and the thyroid, which is a feasible dose optimization scheme.

## Data Availability Statement

The datasets presented in this study can be found in online repositories. The names of the repository/repositories and accession number(s) can be found below: https://www.researchdata.org.cn (RDD number: RDDA2021001995).

## Ethics Statement

Our study was reviewed and approved by the IRB committee of Sun Yat-sen University Cancer Center, with the approval number of B2021-234. As this study is only a retrospective analysis, informed consent exemption was approved.

## Author Contributions

XD and CZ contributed conception and design of the study. GS, YP, JL, HW, and GZ organized the database. YP, JL, HW, and GZ performed the statistical analysis. GS wrote the first draft of the manuscript. GS and YP wrote sections of the manuscript. All authors contributed to the article and approved the submitted version.

## Funding

This work was jointly supported by National Natural Science Foundation of China (12005316), and Cancer Precision Radiotherapy Spark Program of China International Medical Foundation (2019-N-11-20).

## Supplementary Material

The Supplementary Material for this article can be found online at: https://www.frontiersin.org/articles/10.3389/fonc.2021.714536/full#supplementary-material

## Conflict of Interest

The authors declare that the research was conducted in the absence of any commercial or financial relationships that could be construed as a potential conflict of interest.

## Publisher’s Note

All claims expressed in this article are solely those of the authors and do not necessarily represent those of their affiliated organizations, or those of the publisher, the editors and the reviewers. Any product that may be evaluated in this article, or claim that may be made by its manufacturer, is not guaranteed or endorsed by the publisher.
