# Supplementary material for: Multivariate NTCP Model of Hypothyroidism After Intensity-Modulated Radiotherapy for Nasopharyngeal Carcinoma
Source: Front Oncol. 2021 Aug 23;11:714536. doi: 10.3389/fonc.2021.714536 (PMC8421234; doi:10.3389/fonc.2021.714536)
Supplement: Supplementary file 1 [file DataSheet_1.docx]

**Table. Spearman's correlation analysis**

|  |  |  | Age | Dmin | Dmean | V20 | V30 | V40 | V45 | V50 | Volume |
| --- | --- | --- | --- | --- | --- | --- | --- | --- | --- | --- | --- |
| Spearman's rho | Age | Correlation Coefficient | 1.000 | -.233 | -.233 | -.220 | -.213 | -.236 | -.164 | -.123 | .321^**^ |
|  |  | Sig. (2-tailed) | . | .054 | .054 | .069 | .079 | .051 | .179 | .313 | .007 |
|  |  | N | 69 | 69 | 69 | 69 | 69 | 69 | 69 | 69 | 69 |
|  | Dmin | Correlation Coefficient | -.233 | 1.000 | .856^**^ | .901^**^ | .951^**^ | .843^**^ | .765^**^ | .684^**^ | -.301^*^ |
|  |  | Sig. (2-tailed) | .054 | . | .000 | .000 | .000 | .000 | .000 | .000 | .012 |
|  |  | N | 69 | 69 | 69 | 69 | 69 | 69 | 69 | 69 | 69 |
|  | Dmean | Correlation Coefficient | -.233 | .856^**^ | 1.000 | .896^**^ | .915^**^ | .973^**^ | .965^**^ | .922^**^ | -.272^*^ |
|  |  | Sig. (2-tailed) | .054 | .000 | . | .000 | .000 | .000 | .000 | .000 | .024 |
|  |  | N | 69 | 69 | 69 | 69 | 69 | 69 | 69 | 69 | 69 |
|  | V20 | Correlation Coefficient | -.220 | .901^**^ | .896^**^ | 1.000 | .915^**^ | .870^**^ | .809^**^ | .721^**^ | -.248^*^ |
|  |  | Sig. (2-tailed) | .069 | .000 | .000 | . | .000 | .000 | .000 | .000 | .040 |
|  |  | N | 69 | 69 | 69 | 69 | 69 | 69 | 69 | 69 | 69 |
|  | V30 | Correlation Coefficient | -.213 | .951^**^ | .915^**^ | .915^**^ | 1.000 | .916^**^ | .852^**^ | .770^**^ | -.251^*^ |
|  |  | Sig. (2-tailed) | .079 | .000 | .000 | .000 | . | .000 | .000 | .000 | .038 |
|  |  | N | 69 | 69 | 69 | 69 | 69 | 69 | 69 | 69 | 69 |
|  | V40 | Correlation Coefficient | -.236 | .782^**^ | .973^**^ | .870^**^ | .916^**^ | 1.000 | .963^**^ | .902^**^ | -.285^*^ |
|  |  | Sig. (2-tailed) | .051 | .000 | .000 | .000 | .000 | . | .000 | .000 | .018 |
|  |  | N | 69 | 69 | 69 | 69 | 69 | 69 | 69 | 69 | 69 |
|  | V45 | Correlation Coefficient | -.164 | .765^**^ | .965^**^ | .809^**^ | .852^**^ | .963^**^ | 1.000 | .969^**^ | -.285^*^ |
|  |  | Sig. (2-tailed) | .179 | .000 | .000 | .000 | .000 | .000 | . | .000 | .017 |
|  |  | N | 69 | 69 | 69 | 69 | 69 | 69 | 69 | 69 | 69 |
|  | V50 | Correlation Coefficient | -.123 | .684^**^ | .922^**^ | .721^**^ | .770^**^ | .902^**^ | .969^**^ | 1.000 | -.233 |
|  |  | Sig. (2-tailed) | .313 | .000 | .000 | .000 | .000 | .000 | .000 | . | .054 |
|  |  | N | 69 | 69 | 69 | 69 | 69 | 69 | 69 | 69 | 69 |
|  | Volume | Correlation Coefficient | .321^**^ | -.301^*^ | -.272^*^ | -.248^*^ | -.251^*^ | -.285^*^ | -.285^*^ | -.233 | 1.000 |
|  |  | Sig. (2-tailed) | .007 | .012 | .024 | .040 | .038 | .018 | .017 | .054 | . |
|  |  | N | 69 | 69 | 69 | 69 | 69 | 69 | 69 | 69 | 69 |

Note: * P < 0.05; ** P < 0.01, using Spearman's correlation analysis
